# Two Cases of Methimazole-Induced Agranulocytosis With Their Risk Factors

**DOI:** 10.1016/j.aace.2021.10.005

**Published:** 2021-11-03

**Authors:** Aye Khine, Kiranjit Dhillon, Linda Jo, Vanessa Wormser, Soe Naing, Shreela Mishra

**Affiliations:** 1 Department of Internal Medicine, University of California San Francisco-Fresno, Fresno, California; 2 Department of Medicine, Division of Endocrinology, University of California San Francisco-Fresno, Fresno, California

**Keywords:** agranulocytosis, antithyroid, hyperthyroidism, methimazole, ANC, absolute neutrophil count, Hgb, hemoglobin, MMI, methimazole, MMI-AGRAN, MMI-induced agranulocytosis, Plt, platelet, T3, triiodothyronine, T4, thyroxine, TSH, thyroid-stimulating hormone, WBC, white blood count

## Abstract

**Background:**

Antithyroid drugs, such as methimazole (MMI), are standard therapies for the medical management of thyrotoxicosis. Agranulocytosis is a rare but lethal adverse effect of antithyroid medications. We have reported 2 cases of MMI-induced agranulocytosis with similar risk factors that likely predisposed them to this adverse reaction.

**Case Report:**

Case 1 involved a 71-year-old woman, with a history of Graves disease, who presented with an altered mental status. She was recently discharged on 40 mg of MMI twice daily, and she continued this dose for 2 months. She was readmitted and found to have neutropenic fever in the setting of MMI-induced agranulocytosis. MMI was discontinued, and she was started on filgrastim. Her cell counts gradually improved, and she was subsequently discharged.

Case 2 involved a 68-year-old woman, with a history of Graves disease, who presented with severe back pain, nausea, and vomiting. She was recently discharged on 10 mg of MMI twice daily, which was increased to 10 mg 3 times a day. She was readmitted to the hospital because of a septic shock in the setting of pneumonia, colitis, bacteremia, and MMI-induced agranulocytosis. A bone marrow biopsy showed a polyclonal infiltrate with up to 85% plasma cells. Despite treatment with antibiotics, filgrastim, and continuous renal replacement therapy, she ultimately passed away.

**Discussion:**

Although these cases had differing outcomes, they shared similar features and risk factors, including older age, female sex, and relatively higher doses of MMI.

**Conclusion:**

Close follow up and awareness of risk factors, such as age, female sex, and higher doses of MMI, may decrease the risk of MMI-induced agranulocytosis and fatal outcomes.

## Introduction

Graves disease and toxic multinodular goiter are 2 of the most common causes of thyrotoxicosis. Treatment primarily consists of antithyroid medications, radioiodine, and surgery. Methimazole (MMI) is a standard drug used for the treatment for Graves disease and toxic multinodular goiter because of its longer duration of action and lower incidence of side effects. Despite MMI’s availability and widespread use, MMI-induced agranulocytosis (MMI-AGRAN) is a rare but potentially deadly side effect that should be noted. The frequency of MMI-AGRAN ranges from 0.2% to 0.5%.^1^ Studies have shown that it typically develops within the first 3 months of drug initiation.^1^ Given the severity of this reaction, close vigilance is necessary. We have described 2 cases of MMI-AGRAN with similar predisposing factors, including a recent increase in the dose of MMI.

## Case Report

### Case 1

A 71-year-old woman with a past medical history of recently diagnosed Graves disease presented to the emergency room with an altered mental status for 3 days. She had been recently hospitalized 3 months prior for a thyroid storm in the setting of newly diagnosed Graves disease with a thyroid-stimulating hormone (TSH) level of <0.01 μ[IU]/mL (0.45–5.33 μ[IU]/mL), a free thyroxine (T4) level of 3.51 ng/dL (0.89–1.76 ng/dL), a free triiodothyronine (T3) level of 6.6 pg/mL (2.3–4.2 pg/mL), and thyroid-stimulating immunoglobulin positivity. She was started on 40 mg of MMI twice daily and discharged on this dose because of a persistent elevation of free T4 at 3.01 ng/dL (0.89–1.76 ng/dL) on the day of discharge. She was also discharged with 60 mg of propranolol twice daily. Her laboratory test results on the day of discharge were notable for a white blood cell (WBC) count of 12.7 × 10^3^/μL (4.0 × 10^3^-11.0 × 10^3^/μL), hemoglobin (Hgb) level of 9.0 g/dL (12-16 g/dL), and platelet (Plt) count of 293 × 10^3^/μL (140 × 10^3^-440 × 10^3^/μL). The patient was scheduled for outpatient endocrinology follow up to taper MMI, but she missed her appointment because of a humerus fracture. She continued to receive refills of MMI through her primary care provider for over 2 months, until she developed an altered mental status and presented again to the emergency department.

In the emergency room, her blood pressure was 107/66 mm Hg, with a heart rate of 77 beats/min, respiratory rate of 18 breaths/min, temperature of 39.5°C, and oxygen saturation of 96% on room air. Physical examination did not reveal any thyromegaly, thyroid nodules, or goiter. Her laboratory test results were remarkable for a WBC count of 0.5 × 10^3^/μL (4.0 × 10^3^-11.0 × 10^3^/μL), absolute neutrophil count (ANC) of 0.0 × 10^3^/μL (1.6 × 10^3^-7.3 × 10^3^/μL), Hgb level of 9.0 g/dL (12-16 g/dL), Plt count of 293 × 10^3^/μL (140 ×10^3^-440 × 10^3^/μL), TSH level of 0.365 μ[IU]/mL (0.45-5.33 μ[IU]/mL), and free T4 level of 0.37 ng/dL (0.89-1.76 ng/dL). MMI and propranolol were held on admission. A workup for infection revealed stool study results positive for a *Clostridium difficile* toxin in the setting of diarrhea; therefore, she was treated with oral vancomycin. Her mental status gradually improved. By day 6 of admission, her ANC was still 0.0 × 10^3^/μL (1.6 × 10^3^-7.3 × 10^3^/μL); she was started on 300 μg of filgrastim daily. By day 10, her ANC was 0.2 × 10^3^/μL (1.6 × 10^3^-7.3 × 10^3^/μL), and she was discharged on a 10-day course of filgrastim. She was also restarted on 60 mg of propranolol twice daily.

Two weeks after discharge, her ANC was 1.9 × 10^3^/μL (1.6 × 10^3^-7.3 × 10^3^/μL). However, she was experiencing night sweats, increased hunger, and anxiety. Laboratory tests showed a TSH level 0.166 μ[IU]/mL (0.45-5.33 μ[IU]/mL), free T3 level of 5.7 pg/mL (2.34.2 pg/mL), and free T4 level of 1.34 ng/dL (0.89-1.76 ng/dL). She was continued on 60 mg of propranolol twice daily. She was eventually started on potassium iodide and 0.3 mL of iodine (Lugol solution) 3 times daily, and she later underwent thyroidectomy. After the thyroidectomy, her symptoms resolved. She developed postsurgical hypothyroidism and was placed on 100 μg of levothyroxine daily, with normalization of the TSH level to 3.04 μ[IU]/mL (0.45-5.33 μ[IU]/mL).

### Case 2

A 68-year-old woman with a past medical history of recently diagnosed Graves disease presented to the emergency room with severe back pain, nausea, and vomiting. She had been recently hospitalized 2 months prior and was found to have newly diagnosed Graves disease, with a TSH level of <0.010 μ[IU]/mL (0.45-5.33 μ[IU]/mL), a free T4 level of 2.2 ng/dL (0.89-1.7 ng/dL), a free T3 level of 2.6 pg/mL (2.3-4.2 pg/mL), and thyroid-stimulating immunoglobulin positivity. She was started on 10 mg of MMI twice daily by the inpatient endocrinology department. Laboratory test results on the day of discharge were notable for a WBC count of 8.8 × 10^3^/μL (4.0 × 10^3^-11.0 × 10^3^/μL), Hgb level of 9.9 g/dL (12-16 g/dL), and Plt count of 258 × 10^3^/μL (140 × 10^3^-440 × 10^3^/μL). Her MMI dose was increased to 10 mg 3 times a day by her primary care provider 1 month later. She continued to take this increased dose for 1 month, before presenting again to the emergency department.

In the emergency room, her blood pressure was 85/49 mm Hg, with a heart rate of 102 beats/min, respiratory rate of 20 breaths/min, temperature of 36.7°C, and oxygen saturation of 86% on room air. Physical examination was remarkable for moderate distress but no thyromegaly, thyroid nodules, or goiter. She was admitted to the intensive care unit because of a septic shock and respiratory failure requiring intubation. Her laboratory tests were notable for a WBC count of 0.7 × 10^3^/μL (4.0 × 10^3^-11.0 × 10^3^/μL), ANC of 0.0 × 10^3^/μL (1.6 × 10^3^-7.3 × 10^3^/μL), Hgb level of 8.2 g/dL (12-16 g/dL), and Plt count of 129 × 10^3^/μL (140 ×10^3^-440 × 10^3^/μL). MMI was held on admission. She was started on 300 μg of filgrastim daily. Imaging revealed a new infiltrate in the right lung and suspicion for colitis. She was started on vancomycin and cefepime. Her blood cultures were positive for *Escherichia coli* and pseudomonas. Because of persistent pancytopenia, she underwent a bone marrow biopsy on day 8, which showed a polyclonal infiltrate with up to 85% plasma cells. She underwent bronchoscopy, which showed a fungating mass with numerous fungal organisms, including *Aspergillus fumigatus*; therefore, she was started on amphotericin B. Additionally, her fungal blood cultures were positive for *Rhizomucor pusillus*. Her neutropenia resolved and the WBC count improved to 2.1 × 10^3^/μL (4.0 × 10^3^-11.0 × 10^3^/μL) by day 12. However, she became anuric and was started on continuous renal replacement therapy on day 13. She ultimately passed away on day 14 because of her overwhelming infections and multiorgan failure.

## Discussion

MMI-AGRAN is a rare but life-threatening adverse effect. The mortality rates are as high as 21.5%.^2^ It is imperative to identify risk factors and closely monitor patients taking MMI. The 3 risk factors associated with MMI-AGRAN are increased age, female sex, and higher doses of MMI.^3^, ^4^, ^5^, ^6^, ^7^

Elderly patients may be more prone to developing MMI-AGRAN because of impaired drug metabolism, increased sensitivity to drugs, and polypharmacy leading to adverse drug interactions. However, it is difficult to draw specific conclusions because the exact cause of MMI-AGRAN is unknown.^3^ An early study noted an increased risk of MMI-AGRAN in patients over the age of 40 years. However, there were confounding factors, including a preponderance of women in all 3 groups studied.^3^ Another study demonstrated increased mortality rates in patients over the age of 60 years with agranulocytosis associated with various drugs, including antibiotics, antithyroid drugs (particularly carbimazole), antiepileptics, and antiplatelet agents.^4^

Another risk factor that may be associated with MMI-AGRAN is female sex. One study of 754 cases of MMI-AGRAN had a male-to-female ratio of 1:6.3.^5^ In both the cases described above, the patients were women over the age of 65 years, which might have increased their risk of developing MMI-AGRAN. However, 1 confounding factor might have been that there is a preponderance of women with thyroid disorders and, therefore, a preponderance of women on antithyroid medications, such as MMI, which might have accounted for the increased risk of MMI-AGRAN.

Although we cannot control a patient’s age or sex, some other risk factors can be modified. Studies have shown that there is, likely, a relationship between the dose of MMI and the prevalence of MMI-AGRAN.^5^, ^6^ One study that analyzed 754 cases of antithyroid drug-induced agranulocytosis (725 patients were treated with MMI, 28 patients with propylthiouracil, and 1 patient with both MMI and propylthiouracil) found that an MMI dose of 25.2 ± 12.8 mg/d increased the risk of MMI-AGRAN and was also the dose most associated with death due to MMI-AGRAN. Additionally, a dose of 15 mg/d was found to decrease the prevalence of agranulocytosis.^5^ Another study was performed in a group of 514 patients, in which 144 patients received an initial dose of 30 mg of MMI daily, 44 patients received 20 mg daily, and 277 patients received 15 mg daily. There was a statistically significant difference in the incidence of agranulocytosis between patients who received 30 mg daily and those who received 15 mg daily, suggesting that the incidence of agranulocytosis with low-dose MMI (15 mg daily or lower) was 10 times lower than that in those who received high-dose MMI (20 mg daily or higher).^6^ Furthermore, a prospective randomized trial comparing different doses of MMI concluded that a single daily dose of 15 mg controls Graves hyperthyroidism in most patients and that TSH-binding inhibitor immunoglobulin levels decrease with this regimen in a similar way as that while using the conventional divided dose of 10 mg 3 times daily.^7^ This further supports the concept that the risks of using higher doses of MMI likely far outweigh the benefits.

The 2018 European Thyroid Association guidelines recommend an initial dose of 10 to 30 mg of MMI once daily depending on the severity of hyperthyroidism. The dose can be gradually reduced as thyrotoxicosis improves, titrating based on free T4 and free T3 levels. The usual daily maintenance doses range from 2.5 to 10 mg.^8^ In comparison, the 2016 American Thyroid Association guidelines have a weak recommendation for an initial MMI dose of 10 to 30 mg daily, which is then titrated down to a maintenance dose of 5 to 10 mg daily. They have a rough guide for initial MMI daily dosing: 5 to 10 mg if the free T4 level is 1 to 1.5 times the upper limit of normal, 10 to 20 mg if the free T4 level is 1.5 to 2 times the upper limit of normal, and 30 to 40 mg if the free T4 level is 2 to 3 times the upper limit of normal. MMI is usually given as a once-daily dosing, but an initial split dose of a twice-daily dosing may be beneficial when more rapid biochemical control is needed.^9^ Both the European Thyroid Association and American Thyroid Association have stated that MMI-AGRAN is more likely to occur in the first 3 months of initiating therapy, and therefore, close monitoring is especially needed during this time.^8^^,^^9^

## Conclusion

Although antithyroid medications, such as MMI, are commonly used, more research is needed on their adverse effects and predisposing factors. Lower doses of MMI should be used whenever possible to potentially decrease the risk of developing agranulocytosis and death due to agranulocytosis. Patients should be monitored closely, and MMI doses should be tapered as thyroid function levels improve, especially because an increased MMI dosage does not clearly have increased efficacy.
